# The POZ/BTB and AT-Hook Containing Zinc Finger 1 (PATZ1) Transcription Regulator: Physiological Functions and Disease Involvement

**DOI:** 10.3390/ijms18122524

**Published:** 2017-11-24

**Authors:** Monica Fedele, Elvira Crescenzi, Laura Cerchia

**Affiliations:** CNR—Institute of Experimental Endocrinology and Oncology (IEOS), 80131 Naples, Italy; e.crescenzi@ieos.cnr.it (E.C.); cerchia@unina.it (L.C.)

**Keywords:** PATZ1, chromatin regulator, cancer, biomarker, stem cells

## Abstract

PATZ1 is a zinc finger protein, belonging to the POZ domain Krüppel-like zinc finger (POK) family of architectural transcription factors, first discovered in 2000 by three independent groups. Since that time accumulating evidences have shown its involvement in a variety of biological processes (i.e., embryogenesis, stemness, apoptosis, senescence, proliferation, T-lymphocyte differentiation) and human diseases. Here we summarize these studies with a focus on the PATZ1 emerging and controversial role in cancer, where it acts as either a tumor suppressor or an oncogene. Finally, we give some insight on clinical perspectives using PATZ1 as a prognostic marker and therapeutic target.

## 1. Introduction

**Figure 1:**
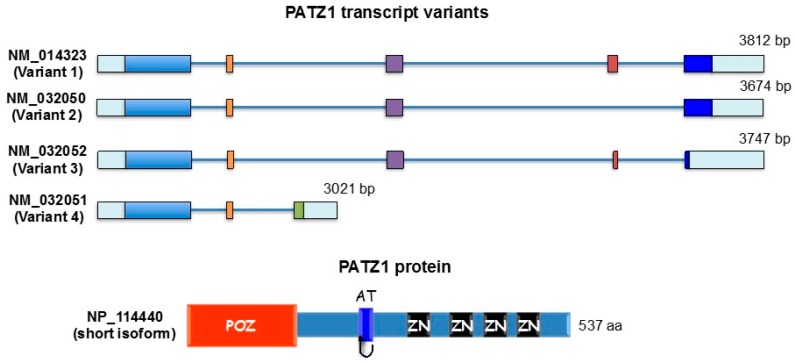
*PATZ1* transcript variants and protein structure. Schematic representation of the 4 transcript variants of the *PATZ1* gene. Variant 1 represents the longest transcript and encodes the longest isoform (long C isoform). Variant 2 lacks an alternate segment in the 3′ coding region, compared to variant 1. The resulting protein (long A isoform) maintains the same reading frame and is shorter when compared to the long C isoform. Variant 3 uses an alternate splice site in the 3′ coding region, compared to variant 1, that results in a frameshift. It encodes the long B isoform which has a shorter and distinct C-terminus compared to the long C isoform. Transcript variant 4 differs in the 3′ UTR and has multiple coding region differences (compared to variant 1), one of which results in a frameshift. This results in a shorter protein (short isoform) with a distinct C-terminus, compared to the long C isoform. The schematic protein structure of a representative PATZ1 isoform is shown at the bottom. POZ: POZ/BTB domain; AT: AT-hook domain; ZN: Zinc finger domain.

The POZ/BTB and AT-hook-containing Zinc finger protein 1 (PATZ1), also known as MAZ Related factor (MAZR), Zinc finger Sarcoma Gene (ZSG) or Zinc finger Nuclear Factor/Zinc finger protein 278 (ZNF278/Zfp278), is a transcriptional regulatory factor that has been shown to modulate the expression of different genes either negatively or positively depending on the cellular context [1]. It was first discovered by our group as an interacting protein with the RING finger Nuclear Factor 4 (RNF4) with which it cooperates in gene transcriptional regulation [2]. At the same time, two other laboratories came across the study of this gene while looking for proteins interacting with the B-cell and neuron-specific transcription repressor Bach2, a CNC-related bZip factor [3] and studying an intra-chromosomal rearrangement of chromosome 22 occurring in small round cell sarcoma, in which part of the new ZSG gene was found fused to the Ewing Sarcoma gene (EWS) causing loss of function of ZSG and gain of function of EWS [4]. The *PATZ1* gene, which maps on human chromosome 22q12, includes six exons and gives rise to four splicing transcript variants ranging from 3021 bp (short isoform) to 3812 bp (Long C isoform) (GenBank accession numbers: NM_014323; NM_032050; NM_032052; NM_032051) coding for four protein isoforms ranging from 537 aa (NP_114440; NP_114441) to 641 aa (NP_114439) and 687 aa (NP_055138) [4] (Figure 1). Structurally, all protein variants are characterized by a Poxvirus and Zinc-finger (POZ)/Broad complex, Tramtrack, and Bric à Brac (BTB) domain at the N-terminus, an AT hook DNA binding motif before the first zinc finger, and a cluster of classical Cys_2_-His_2_ zinc fingers (four to six depending on the splicing variant) at the C-terminus [4]. The latter reiterated motif, which presents two conserved cysteine and histidine residue pairs linked to a single zinc ion, is a well known and conserved DNA/RNA binding domain that acts linearly in tandem to allow recognition of nucleic acid sequences of varying length [5]. For this modular organization PATZ1 has been included in a large family of transcription factors, named POZ-ZF or POK, including BCL6, PLZF, TAZ1, and others [6,7], in which the POZ/BTB domain mediates protein-protein interactions, allowing the recruitment of histone deacetylases through co-repressor complexes [7]. Unique in this family of transcription factors is the presence in the PATZ1 protein of an AT-hook, another DNA binding motif that binds DNA through the minor groove, usually cooperating with other DNA binding protein complexes, to play key roles in chromatin-remodeling and transcription regulation [8].

A role for PATZ1 in the regulation of gene expression has been demonstrated in many studies, showing that it acts as either an activator or a repressor depending on the cellular context [1]. As for other members of the POK family, the region N-terminal to the Zn fingers of PATZ1 does not show any transcriptional activity, suggesting that PATZ1 does not act as a typical trans-activator but as an architectural transcription factor [3]. PATZ1 is indeed an interacting protein of Ncor and Sirt1 and modulates histone acetylation levels [9,10]. A similar *modus operandi* is that of the non-histone chromatin-associated HMGA proteins that share with PATZ1 the presence of the AT-hook domain and are involved in the remodeling of the chromatin during transcription, by allowing the access of proteins to DNA, and in the formation of multi-protein complexes on the promoter and enhancer regions of many eukaryotic genes [11]. Differently from HMGA proteins, with which PATZ1 has been shown to co-immunoprecipitate [12], PATZ1 recognizes, through its zinc fingers, a specific binding site consensus, which is GC-rich and highly related to that of MAZ and Sp1, other than an AT-rich sequence possibly through its AT-hook. Therefore, PATZ1 has possibly a double chromatin-remodeling role by virtue of both the POZ/BTB and the AT-hook domains that are also both involved in protein-protein interactions and assembly of multi-protein complexes. The latter feature allows PATZ1 to change regulation of gene transcription from activation to repression and *vice versa*, depending on the interacting proteins and, therefore, the cellular context. For example, Bach2 is a transcriptional repressor, but it turns out to be an activator of transcription when complexed with PATZ1 [3]. On the other hand, the association of PATZ1 with RNF4 switches the activation to repression of selected promoters, including that of the androgen receptor (AR) [13]. Moreover, PATZ1 has been shown to either activate or repress the same promoter in different cellular contexts. This is the case of the *c-myc* promoter that is strongly activated or repressed by PATZ1 in B lymphocytes and cervix carcinoma cells, respectively [2,3], or the case of three p53 target genes, *CDKN1A*, *MDM2*, and *BAX*, whose promoters are activated or repressed depending on the presence/absence of p53, which directly interacts with PATZ1, with consequent opposite functions on cellular apoptosis [1]. Interestingly, PATZ1 can also compete with p53 for the binding to DNA, resulting in an inhibitory function on p53 following DNA damage [14]. Consistently, PATZ1 has also been shown to be an inhibitor of p53-dependent endothelial cell senescence [10]. Collectively, these studies indicate that PATZ1 is a regulator of p53-dependent transcription and, consequently, implicated in cellular processes governed by p53, including DNA damage response, senescence and apoptosis. Like PATZ1, other members of the POK family can act as either repressors or activators of transcription, depending on the cellular context. This is the case of PLZF, a protein implicated in development, cancer and stem cell biology, that usually acts as a transcriptional repressor by recruiting Ncor, SMTR/mSIN3A and HDACs, thus inducing DNA to be tightly compacted and inaccessible to transcription factors [7]. However, in hematopoietic stem/progenitor cells PLZF activates transcription of Eya2, which led to immortalization of these cells [15].

**Figure 2:**
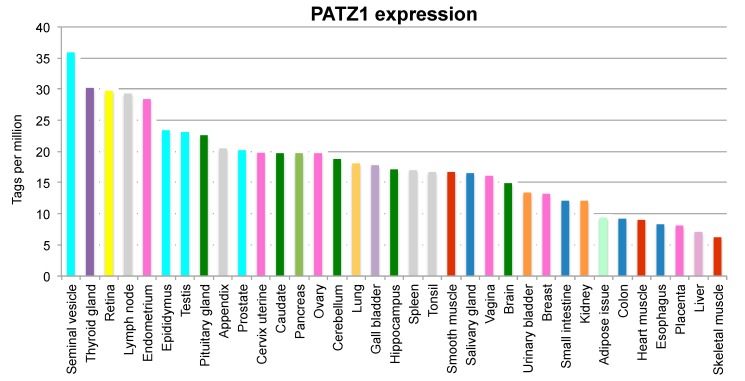
PATZ1 expression in human tissues. mRNA levels of PATZ1 in a panel of human tissues, obtained through Cap Analysis of Gene Expression (CAGE) by the FANTOM5 project [22]. The data have been extracted from the Human Protein Atlas [23]. PATZ1 is expressed at variable levels in all tissues.

PATZ1 is highly and ubiquitously expressed during embryogenesis, especially in the midbrain, where it is restricted to the actively proliferating neuroblasts of the periventricular neocortical neuroepithelium, and is still expressed but at lower levels in all the adult tissues (Figure 2) [3,16], where it appears to be enriched in less differentiated cells [17]. In normal cells it appears mainly nuclear, but either physiological or pathological conditions move its location to the cytosol where it has been shown to interact with the RIalpha regulatory subunit of PKA. However, it is still unknown if this leads to a cytosolic PATZ1 function or is just a way to sequester PATZ1 from its nuclear function as transcription regulator [18]. Only few studies have so far analyzed how PATZ1 expression is regulated. Two of them have focused on its post-transcriptional regulation by microRNAs, showing that miR-29b and miR-24 are specific inhibitors of PATZ1 expression [19,20]. Another study, performed in mouse embryonic stem cells (ESC), demonstrated by ChIP experiments that Oct4 and Nanog pluripotent transcription factors bind to the proximal promoter and the first intron of *Patz1*, respectively, suggesting that *Patz1* is a downstream target of Oct4 and Nanog [21].

## 2. Physiological Functions of PATZ1 and Pathological Implications

Two independent groups have generated Patz1^−/−^ mice by gene targeting approaches, showing that they mostly die in utero or soon after birth likely because of developmental defects in the cardiac outflow tract and/or neural tube closure [9,16]. In Patz1^−/−^ embryos, different malformations of the great vessels that exit from the ventricles of the heart, possibly including their transposition or malposition, were clearly observed. The histological examination of embryos slides suggested an origin of the descending aorta from the right instead of the left primitive dorsal aorta, indicating a role for PATZ1 in left/right determination [15]. This phenotype is reminiscent of the human DiGeorge Syndrome, which hits 1:4000 newborns and is caused by a 22q11-12 chromosomal deletion [24]. One of the genes located in this region and associated with this pathology is TBX1, but other genes are believed to concur to the phenotype, and PATZ1, which is located on 22q12, could be one of them [16]. The other persistent defect in Patz1^−/−^ embryos is in the brain. A significant number of Patz1^−/−^ pups showed exencephaly and hypoproliferation of cells surrounding the brain ventricles, suggesting the involvement of PATZ1 in central nervous system development and possibly in neurogenesis [16]. Consistently, we recently found that Patz1^−/−^ embryos have a reduced number of neural stem cells [25].

### 2.1. Cell Proliferation, Senescence and Apoptosis

The few Patz1^−/−^ mice that reach the adult life, equally distributed between males and females, show a dwarf phenotype likely due to defects in cell proliferation. Indeed Patz1^−/−^ mouse fibroblasts (MEFs) grow slower than wild-type controls [9,16], showing cell cycle defects, premature senescence and increased expression of cell cycle regulators, including both inhibitors (i.e., p53 and the key effector of senescence p16) and activators (i.e., CDK4, cyclin D2, HMGA1/2). These conflicting signals were supposed to be responsible of a hypermitogenic arrest, which in turn induces a premature senescence [16]. The senescent phenotype of Patz1^−/−^ MEFs is likely responsible of the inefficient reprogramming of the Patz1^−/−^ MEFs to iPSC, as detailed below [26]. Downregulation of PATZ1 in human young endothelial cells also induces premature senescence through a ROS-mediated and p53-dependent pathway, contributing to aging associated vascular diseases [10]. Consistent with its role in senescence, *PATZ1* was recently described among the 52 differentially expressed genes that characterize the senescence core signature [27].

Coming back to Patz1-knockout mice, the number of apoptotic cells, spontaneously occurring or resulting after 5-fluorouracil treatment, is reduced in Patz1-null mice compared to wild-type controls, suggesting a proapoptotic role for PATZ1 [1]. However, studies in human cancer cell lines showed opposite results, in which the silencing of *PATZ1* enhances sensitivity to apoptotic stimuli [1,28], and PATZ1 inhibits p53 binding to DNA [14]. This is consistent with the general idea that PATZ1 function is cell context-dependent and may be dependent on the presence of an endogenous wild-type p53 protein [1].

Interestingly, Patz1^+/−^ MEFs do not show premature senescence nor differences in spontaneous apoptosis, but a reduced susceptibility to die following 5-FU treatment and faster growth than wild-type controls has been observed [1,16].

### 2.2. Cell Pluripotency and Reprogramming

Ow et al. demonstrated that PATZ1 is an essential pluripotency regulator of embryonic stem cells (ESCs) [21]. PATZ1 directly regulates the master pluripotency regulators *Pou5f1* and *Nanog* and is part of a sub-network of proteins associated with the establishment and maintenance of pluripotency, including NANOG, OCT4, and DPP4, that have been found to independently interact with PCGF1, a member of the Polycomb Repressor Complex 1 (PRC1) [21,29]. On the other hand, NANOG and the heterodimer OCT4/SOX2 bind and potentially regulate the *PATZ1* gene [30]. Therefore, it has been suggested that PATZ1 is integrated in the transcriptional network that serves to maintain OCT4 and NANOG expression in ESCs [21]. A similar role could also be played by PATZ1 in neural stem cells, as indicated by our studies in Patz1^−/−^ mice, as mentioned above [16], and in cancer stem cells, as suggested by its enriched expression in stem versus non-stem cancer derived cells [31].

On this basis, PATZ1 role in reprogramming of MEFs towards induced pluripotent stem cells (iPSCs), through ectopic expression of the Yamanaka cocktail [32], has been analyzed [26]. As above mentioned, Patz1^−/−^ MEFs gave low reprogramming efficiency likely due to their senescent phenotype. In contrast, Patz1^+/−^ MEFs were more efficiently reprogrammed, and PATZ1 overexpression in wild-type MEFs significantly repressed reprogramming, suggesting that PATZ1 negatively regulates reprogramming and that a critical control of PATZ1 dosage is essential for the generation of iPSCs [26]. In particular, it has been shown that Patz1^+/−^ MEFs display epigenetic histone modifications, including higher levels of acetylated histone H3, H3K4me2, H3K4me3, H3K36me3, and lower levels of histone H3K9me3 and HP1α, that are consistent with a chromatin more accessible for transcriptional activation [26].

### 2.3. Spermatogenesis and Sexual Development

Another defect of the Patz1-null mice is infertility in both females and males. Reduced seminiferous tubules and block of spermatogenesis due to germ cell death for apoptosis was observed in Patz1^−/−^ testes, suggesting that PATZ1 may have a role in the spermatogenic process [17]. Spermatogenesis is essentially dependent on the action of androgens [33]. As above mentioned, PATZ1 is an AR regulator that acts by attenuating the coactivator activity of RNF4 [13]. Therefore, the absence of PATZ1 may alter the regulation of AR machinery, which is crucial for the germ cell maturation, resulting in the activation of apoptotic pathways. A subsequent study of genome-wide association with sexual maturation in human males and females found that a critical polymorphic DNA region, associated with earlier pubertal timing and diminished pubertal growth, harbors a consensus site for PATZ1 and is predicted to affect its binding [34]. Therefore, PATZ1 seems to have a potentially broad effect in the sexual development of both sexes.

### 2.4. T Cell Development

Ellmeier’s group discovered that PATZ1/MAZR is a crucial repressor of CD8 gene expression at the CD4^−^/CD8^−^ double-negative stage of T cell development, by keeping the local chromatin in a condensed state [35]. Consistently, Patz1^−/−^ mice showed higher ratio of CD4^+^ to CD8^+^ cells partially due to redirected differentiation of MHC class I-restricted CD8^+^ single-positive thymocytes into the CD4 helper lineage. Moreover, derepression of ThPOK, an essential transcription factor for the T helper lineage differentiation, was observed in Patz1^−/−^ thymocytes [9]. Subsequently, the complex mechanism of ThPOK dowregulation by PATZ1 was elucidated, showing it acts synergistically with Runx during CD8^+^ T cell lineage development [36]. Therefore, they provided genetic biochemical evidences that PATZ1 is part of the transcription factor network that controls the CD4-versus-CD8 cell-fate “decision” of CD4^+^/CD8^+^ double-positive thymocytes. For a recent and comprehensive review on this complex transcription network see elsewhere [37].

## 3. PATZ1 in Cancer

A growing list of human diseases has involved PATZ1 in their pathogenesis. First among them is cancer, where PATZ1 has been indicated as oncogene, tumor suppressor, or double oncogene/tumor suppressor depending on the tumor type.

### 3.1. Tumor Suppressor Function

The pioneering paper on PATZ1 by Mastrangelo et al. [4] observed that in a small round cell sarcoma *PATZ1*/*ZSG* gene was rearranged on one allele and lost on the other allele, resulting in a complete loss of the wild-type PATZ1 protein expression, thus suggesting it might be a new tumor suppressor gene. In support of this, a subsequent study identified the chromosomal location of *PATZ1* as the human fragile site FRA22B with a likely causative role in the generation of cancer-specific rearrangements [38].

**Figure 3:**
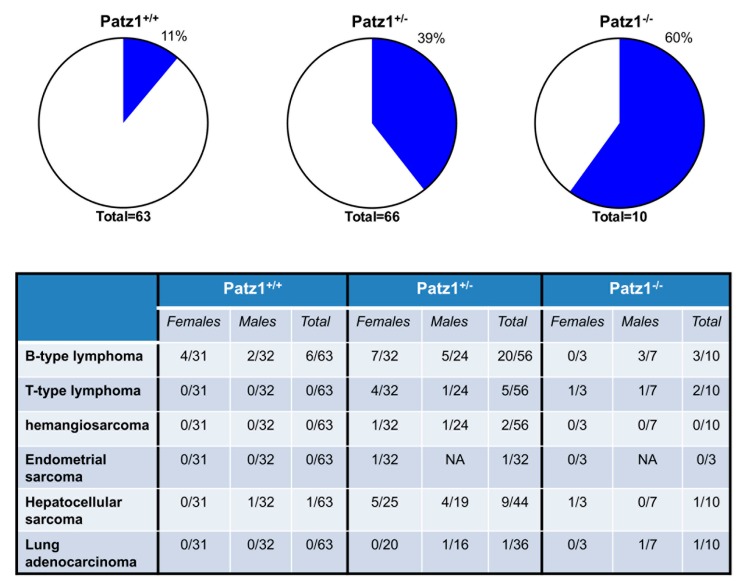
Malignant tumors in PATZ1 knockout mice. Percentage of mice showing malignant tumors are showed in pie charts on the top. The specific tumor phenotype, and relative number of mice showing it, is reported in the table. The mice analyzed were wild-type (Patz1^+/+^) and carrying one (Patz1^+/−^) or two (Patz1^−/−^) knockout allele. NA = not applicable.

The generation of Patz1-knockout mice [16] and the characterization of their phenotype by our group confirmed the tumor suppressor hypothesis since a significant number of both heterozygous and homozygous Patz1-knockout mice developed neoplastic lesions, including lymphomas, hepatocellular carcinomas, and rare sarcomas and lung adenocarcinomas (Figure 3) [39]. The observations in mice are consistent with recent data obtained in some human cancers, where PATZ1 expression is downregulated in malignant versus normal tissues [40,41]. In thyroid cancer, the degree of downregulation further increases proceeding from differentiated to undifferentiated carcinomas, suggesting a PATZ1 role in thyroid cancer progression. Consistently, the restoration of PATZ1 expression in dedifferentiated thyroid cancer cells inhibited their malignant behavior both in vitro, including their capacity to migrate and invade, and in vivo, including a partial re-differentiation [40]. Mechanistically, PATZ1 appears involved in the suppression of the mesenchymal-to-epithelial transition (EMT) through transcriptional regulation of p53 target genes, including *EpCam*, *Caldesmon*, and *RhoE* [40]. In this frame, also a potential role of PATZ1 in thyroid cancer stem cells might be taken in consideration. Recent studies in our laboratory have shown that PATZ1 restoration in rat thyroid cells, transformed by ectopic expression of the Ha-Ras^V12^ oncogene, enhances efficiency of thyrosphere formation but decreases their self-renewal ability [42].

The EMT suppressive role of PATZ1 has been also envisaged for human lung cancer, where PATZ1 expression levels are lower in lymph node metastases than in the primary tumor, and ectopic expression of PATZ1 in A549 lung cancer cells was shown to inhibit migration/invasion in vitro and invasion/colonization in vivo [43]. In this case, the effect of PATZ1 was due to its negative feedback loop on IKK/NF-kB signaling, thus preventing cancer cells from over-stimulation by growth factors or inflammatory mediators, and therefore reducing migration/invasion [43]. The EMT is considered a critical process for the development of metastases and the acquisition of chemoresistance. In this context, blocking EMT might be a possible therapeutic approach and PATZ1 a valuable target to be activated [44]. In other human malignancies, such as Diffuse Large B Cell Lymphomas (DLBCL), the tumor suppressor function of PATZ1 is proapoptotic, achieved by inhibiting and enhancing transcription of BCL6 and BAX, respectively [41]. This is consistent with the already mentioned proapoptotic role of PATZ1 observed in MEFs [1].

Downregulation of PATZ1 expression in thyroid cancer cells has been associated with the activation of the Ras signaling, which leads to overexpression of miR-29b that, in turn, targets and downregulates PATZ1 [19]. However, in thyroid cancer PATZ1 protein is not only downregulated compared to normal thyroid tissue, but it is also increasingly delocalized from the nucleus to the cytoplasm proceeding from differentiated to undifferentiated thyroid carcinomas [40]. A similar situation has been also observed in B cell lymphoma, including Follicular Lymphoma and DLBCL, where the frequency of cells with nuclear expression of PATZ1 was reduced in most of them, together with a cytoplasmic delocalization of the protein [41]. Furthermore, in testicular germ cell tumors the expression of *PATZ1* gene was increased, but the PATZ1 protein was delocalized in the cytoplasm in association with estrogen receptor-β (ERβ) downregulation [17,45]. It is worth of noting that translocation of PATZ1 from cytoplasm to nucleus is induced by cAMP, which also induces increased expression and nuclear localization of ERβ in testicular germ cell tumors [18,45]. In all these cases, we do not know whether a new cytoplasmic function of PATZ1 is gained, but certainly there is a loss of its nuclear, and possibly tumor suppressive, function.

In the nucleus PATZ1 has been described to localize in distinct nuclear dots in association with RNF4 [2], the papilloma virus HPV16 L2 protein [46], and ERβ [45]. Interestingly, these dots are frequently in close association with the PML-oncogenic domains (PODs), which include PML, p53, p73, HIPK2, Daxx, and many other proteins playing fundamental roles in tumor suppression [46,47]. As for other proteins included in these subnuclear structures, the compartmentalization of PATZ1 and the cooperation with binding partners, might decide about its tumor suppressor function.

### 3.2. Oncogenic Function

A potential oncogenic function for PATZ1 has been described in colorectal cancer [48]. Indeed, knockdown of PATZ1 in SW1116 colon cancer cells significantly inhibited cell growth, while overexpression promoted it, by acting at the G1/S cell cycle transition [14,48]. Moreover, the PATZ1 role as an inhibitor of the p53 protein marks it as a proto-oncogene [14].

### 3.3. Double Oncogenic/Tumor Suppressor Function

A double oncogenic/tumor suppressor role might be attributed to PATZ1 in malignant gliomas. A first study showed that silencing of *PATZ1* in GBM cell lines enhanced their sensitivity to chemotherapeutic agents, suggesting PATZ1 might contribute to chemotherapy resistance of GBM [26]. Main contributors of the GBM resistance against the most common therapeutic treatments are the Glioma Stem Cells (GSCs) [49]. A recent study from our group has shown that PATZ1 is overexpressed in a high percentage of GBMs and is enriched in the GSCs, suggesting that, as for its already mentioned role in ESCs [21], it might contribute to the maintenance of the stem cell phenotype and, for this reason, have an oncogenic role [31]. However, when we analyzed survival curves of GBM patients in function of PATZ1 expression, a significant negative correlation between PATZ1 and survival was observed [31]. This is consistent with the enrichment of PATZ1 in the proneural subtype, which has a stem cell signature and is unresponsive to both radio- and chemotherapy, but also has the longest survival among GBM subtypes [50]. However, PATZ1 expression can stratify the proneural subgroup in two distinct clinical subpopulations, where lower levels of *PATZ1* are associated with increased levels of the mesenchymal inducer *CXCR4* and a worst prognosis [31]. Overexpression of PATZ1 in GBM cells causes downregulation of CXCR4 as soon as 48 h after transfection [31]. Therefore, PATZ1 might repress transcription of *CXCR4* avoiding the transdifferentiation towards the more aggressive mesenchymal subtype.

More recently, a comprehensive genomic profiling of pediatric gliomas identified a rearrangement of the *PATZ1* gene (exons 1–5 of *PATZ1* fused downstream to exons 1–9 of *EWSR1*) in a pediatric high-grade glioma [51]. This is the same rearrangement previously identified in a small round cell sarcoma [3], suggesting that the *EWSR1-PATZ1* fusion might be a main oncogenic driver in these tumors.

## 4. PATZ1 in Other Human Pathologies

Tightly associated with the role of PATZ1 in endothelial cell (EC) senescence is its function in diabetes. PATZ1 is upregulated in ECs under diabetic conditions, and elevated endothelial PATZ1 may promote the vascular dysfunction in diabetes. Indeed, PATZ1 overexpression in ECs inhibits angiogenesis and renders ECs unresponsive to angiogenic factors [52]. The mechanism involves the transcription repressive activity of PATZ1 on the fatty acid-binding protein 4 (FABP4), an essential convergence point for angiogenic and metabolic signaling pathways in ECs. Therefore, it has been suggested that PATZ1 serves as a brake on EC differentiation and activation that is normally induced by stimulation with angiogenic ligands [52]. Moreover, PATZ1 is expressed at lower levels in the endothelium of vascular smooth muscle cells of atherosclerotic tissue [10], and has been found coexpressed with TGFB1 in human atherosclerotic carotid tissue [53]. Therefore, PATZ1 has been proposed to be involved in fine-tuning of *TGFB1* expression in atherosclerosis [53].

The analysis of gene expression patterns through computational methods has allowed the discovery that PATZ1 is linked to other two common human disorders: depression and osteoarthritis [54,55]. Depression has been recently classified into at least two subtypes: major depressive disorder (MDD) and subsyndromal symptomatic depression (SSD). PATZ1 is one of the key differentially regulated factors in SSD, suggesting it may have a role in this transitory depressive state [54]. This hypothesis is consistent with a PATZ1 role in neurogenesis. Indeed, treatments against depression increase neurogenesis and, when neurogenesis is suppressed, antidepressant are no longer effective [56].

Also in Osteoarhtritis, the most common form of arthritis, PATZ1 has been found as one of the top ranked transcription factors of its regulatory network, suggesting a potential role in its initiation [55].

Interestingly, it has been recently shown that PATZ1 downregulates the expression of FADS1, a key enzyme in the biosynthesis of long-chain polyunsaturated fatty acids (LC-PUFA) [57]. An excess of LC-PUFA may be responsible of chronic inflammation and numerous inflammatory diseases, including atherosclerosis, arthritis, cancer, and determinant in neuron growth and brain development [57].

## 5. Final Remarks and Clinical Perspectives

A recent study found PATZ1 among the top ten transcriptional factors strongly associated with the renal cell carcinoma (RCC), indicating that PATZ1 might be used as biomarker of RCC for accurate diagnosis, prognosis and/or treatment efficiency [58]. Similarly, as mentioned above, in GBMs and DLBCLs patients there are evidences that PATZ1 lower levels predict a worst survival [31,41]. In GBM it could be particularly helpful to further characterize the still heterogeneous proneural sub-type where low levels of PATZ1 correlate with worse outcome in both overall and progression-free survival curves, independently from other known prognostic factors, such as MGMT promoter methylation and age of diagnosis [31]. Also in DLBCLs, PATZ1 appears to have a prognostic value independent from other known prognostic factors, such as IPI code and cell of origin, in patients treated with rituximab plus cyclophosphamide, doxorubicin, vincristine and prednisone chemotherapy [41].

Importantly, PATZ1 expression is downregulated, possibly at post-transcriptional level, in response to the DNA damage-inducing drug doxorubicin [14]. Therefore, since PATZ1 downregulation is predictive of a worst prognosis, we could speculate that PATZ1 is responsible of a positive effect of chemotherapy on tumor exacerbation. This would highlight the need for searching the negative regulators of PATZ1 involved in this response and use them as valuable targets for an adjuvant cancer therapy.

In conclusion, there is no doubt that PATZ1 plays central roles in a wide range of physiologic functions and pathological conditions. In many cases, especially in cancer, its role depends on the cellular context and it can either enhance or counteract the neoplastic process. However, much study is still needed on the molecular mechanisms upstream and downstream of this important zinc finger protein.

## Abbreviations

POK	POZ domain Krüppel-like zinc finger
CNC	Cap’n’collar
bb	Base pairs
aa	amino acids
IPI	International Proliferation Index
